# Antipsychotic prescription, assumption and conversion to psychosis: resolving missing clinical links to optimize prevention through precision

**DOI:** 10.1038/s41537-022-00254-8

**Published:** 2022-05-04

**Authors:** TianHong Zhang, Andrea Raballo, JiaHui Zeng, RanPiao Gan, GuiSen Wu, YanYan Wei, LiHua Xu, XiaoChen Tang, YeGang Hu, YingYing Tang, HaiChun Liu, Tao Chen, ChunBo Li, JiJun Wang

**Affiliations:** 1 Shanghai Mental Health Center, Shanghai Jiaotong University School of Medicine, Shanghai Intelligent Psychological Evaluation and Intervention Engineering Technology Research Center (20DZ2253800), Shanghai Key Laboratory of Psychotic Disorders, Shanghai, 200030 People’s Republic of China; 2 Department of Medicine, Section of Psychiatry, Clinical Psychology and Rehabilitation, University of Perugia, Perugia, Italy; 3 Center for Translational, Phenomenological and Developmental Psychopathology (CTPDP), Perugia University Hospital, Perugia, Italy; 4 Department of Automation, Shanghai Jiao Tong University, Shanghai, 200240 China; 5 Big Data Research Lab, University of Waterloo, Waterloo, ON Canada; 6 Senior Research Fellow, Labor and Worklife Program, Harvard University, Cambridge, MA USA; 7 CAS Center for Excellence in Brain Science and Intelligence Technology (CEBSIT), Chinese Academy of Science, Shanghai, People’s Republic of China; 8 Institute of Psychology and Behavioral Science, Shanghai Jiao Tong University, Shanghai, People’s Republic of China

**Keywords:** Psychosis, Schizophrenia

## Abstract

The current concept of clinical high-risk(CHR) of psychosis relies heavily on “below-threshold” (i.e. attenuated or limited and intermittent) psychotic positive phenomena as predictors of the risk for future progression to “above-threshold” positive symptoms (aka “transition” or “conversion”). Positive symptoms, even at attenuated levels are often treated with antipsychotics (AP) to achieve clinical stabilization and mitigate the psychopathological severity. The goal of this study is to contextually examine clinicians’ decision to prescribe AP, CHR individuals’ decision to take AP and psychosis conversion risk in relation to prodromal symptoms profiles. CHR individuals (*n* = 600) were recruited and followed up for 2 years between 2016 and 2021. CHR individuals were referred to the participating the naturalistic follow-up study, which research procedure was independent of the routine clinical treatment. Clinical factors from the Structured Interview for Prodromal Syndromes (SIPS) and global assessment of function (GAF) were profiled via exploratory factor analysis (EFA), then the extracted factor structure was used to investigate the relationship of prodromal psychopathology with clinicians’ decisions to AP-prescription, CHR individuals’ decisions to AP-taking and conversion to psychosis. A total of 427(71.2%) CHR individuals were prescribed AP at baseline, 532(88.7%) completed the 2-year follow-up, 377(377/532, 70.9%) were taken AP at least for 2 weeks during the follow-up. EFA identified six factors (Factor-1-Negative symptoms, Factor-2-Global functions, Factor-3-Disorganized communication & behavior, Factor-4-General symptoms, Factor-5-Odd thoughts, and Factor-6-Distorted cognition & perception). Positive symptoms (Factor-5 and 6) and global functions (Factor-2) factors were significant predictors for clinicians’ decisions to AP-prescription and CHR individuals’ decisions to assume AP, whereas negative symptoms (Factor-1) and global functions (Factor-2) factors predicted conversion. While decisions to AP-prescription, decisions to AP-taking were associated to the same factors (positive symptoms and global functions), only one of those was predictive of conversion, i.e. global functions. The other predictor of conversion, i.e. negative symptoms, did not seem to be contemplated both on the clinician and patients’ sides. Overall, the findings indicated that a realignment in the understanding of AP usage is warranted.

## Introduction

Operational criteria Clinical high risk (CHR) for psychosis and the related major outcome, i.e. “conversion” or “transition” to psychosis, are the central compass of contemporary early detection strategies and have progressively spread around the world. Despite CHR concept provides a golden window for early prevention and intervention for psychosis, achieving timely, optimal and effective intervention for CHR individuals is still a problematic target. For example, although only less than 30% of CHR convert to psychosis within the following 2 years^1,2^ (and even less among children and adolescents^3,4^), and AP exposure already at inception is relatively common in this clinical population^5–7^.

### Conceptual analysis and empirical questions

Rather than an erratic phenomenon, AP prescription in this population is presumably guided by pondered clinical decisions, yet it is not clear what are the main reasons for such a choice given that CHR are by definition below the clinical threshold for psychosis and related indicated pharmacological treatment^8^. One of the most plausible reasons is that clinicians and treating staff ascribe to these individuals a higher likelihood to convert to psychosis in the near future. A recent meta-analysis, indeed, revealed that AP prescription since baseline in CHR samples is associated with a higher imminent risk of conversion to psychosis, and therefore should be regarded as a non-negligible red flag for clinical risk management^7^. Thus the next logical question is whether these CHR individuals with ongoing AP prescription at enrollment exhibit specific features that make clinicians feel that they are at greater risk. On the other side, i.e. from the perspective of CHR individuals, what are the main reasons to begin AP assumption? Even more importantly, if clinicians or CHR individuals both opt for AP treatment as a necessary therapeutic step during CHR phase, is such decision really consistent with the alleged main symptomatic risk factors leading to first episode psychosis? If these problematic issues are not clarified, they are likely to lead to the repeated occurrence of some empirical errors, and ultimately affect the effect of early intervention.

### Aim of the study

The current study examined whether the factors that are associated to clinicians to prescribe AP to CHR individuals, the factors that are associated to CHR individuals to decide to take AP, and the factors that predict the onset of psychosis are similar. According to our previous findings, which suggest that AP may not be effective in preventing psychosis among CHR youth, we speculate that there may be a misplacement of targets in clinicians’ decisions on prescribing AP, CHR individuals’ decisions on taking AP and determining the progression of psychosis.

## Results

### Sample characteristics

Baseline sample characteristics are summarized in Table 1. There were significant differences between those who did and did not convert on gender, GAF scores, positive, negative and disorganization symptoms.

**Table 1:** Baseline demographic and SIPS variables, comparison between converted-CHR and not-converted-CHR.

Variables	Total sample	Converted-CHR	Not-Converted-CHR	Comparison		Lost
				*t/χ*^*2*^	*P* value	
Cases (*n*)	600	111	421	—	—	68
*Demographic variables*						
Age(years)[mean(S.D.)]	20.4 (6.1)	19.8 (5.6)	20.3 (6.0)	*t* = 0.710	0.478	22.4 (7.5)
Male[*n*(%)]	284 (47.3)	63 (56.8)	191 (45.4)	***χ***^***2***^ = 4.566	**0.033**	30 (44.1)
Female[*n*(%)]	316 (52.7)	48 (43.2)	230 (54.6)			38 (55.9)
Education(years)[mean(S.D.)]	11.3 (3.1)	10.8 (2.6)	11.3 (3.1)	*t* = 1.627	0.104	11.9 (3.5)
Family history(none)[*n*(%)]	477 (79.5)	94 (84.7)	334 (79.3)	***χ***^***2***^ = 2.686	0.261	49 (72.1)
Family history(low-risk),[*n*(%)]	64 (10.7)	7 (6.3)	49 (11.6)			8 (11.8)
Family history(High-risk),[*n*(%)]	59 (9.8)	10 (9.0)	38 (9.0)			11 (16.2)
*SIPS variables*						
APSS,[*n*(%)]	559 (93.2)	105 (94.6)	394 (93.6)	***χ***^***2***^ = 2.084	0.353	60 (88.2)
GRDS,[*n*(%)]	61 (10.2)	13 (11.7)	37 (8.8)			11 (16.2)
BIPS,[*n*(%)]	20 (3.3)	6 (5.4)	12 (2.9)			2 (2.9)
Before GAF[mean(S.D.)]	78.8 (4.4)	78.9 (3.5)	78.6 (4.5)	*t* = 0.552	0.581	79.6 (4.6)
Now GAF[mean(S.D.)]	55.9 (7.3)	53.6 (5.7)	56.1 (7.2)	*t* = 3.373	**0.001**	58.5 (8.8)
GAF drop[mean(S.D.)]	22.9 (7.2)	25.3 (6.0)	22.5 (7.1)	*t* = 3.762	**<0.001**	21.1 (8.7)
Positive symptoms [Mean(S.D.)]	9.3 (3.9)	10.5 (3.5)	9.2 (3.9)	*t* = 3.235	**0.001**	8.2 (4.3)
Negative symptoms [Mean(S.D.)]	11.7 (5.9)	13.8 (6. 1)	11.4 (5.6)	*t* = 3.947	**<0.001**	10.8 (6.3)
Disorganization symptoms [Mean(S.D.)]	5.8 (3.2)	6.4 (3.2)	5.7 (3.1)	*t* = 2.106	**0.036**	5.2 (3.8)
General symptoms [Mean(S.D.)]	9.0 (3.1)	8.7 (3.1)	9.1 (3.1)	*t* = 1.282	0.201	8.6 (3.0)
SOPSTAL [Mean(S.D.)]	35.8 (11.1)	39.3 (11.1)	35.4 (10.6)	*t* = 3.492	**0.001**	32.9 (12.7)

Note: GAF drop, GAF (Global Assessment of Functioning) score baseline from highest in the past year; low-risk family history, having any family members with mental disorders or a first-degree relative with non-psychotic disorders; high-risk family history, having at least one first-degree relative with psychosis; APSS, attenuated positive symptom syndrome; GRDS, genetic risk and deterioration syndrome; BIPS, brief intermittent psychotic syndrome; *t/χ*^*2*^: *t* for independent t test, *χ*^*2*^ for kappa test.

*p* values that are statistically significant are shown in bold.

### Factor extraction

The exploratory factor analysis of 19 SIPS items and two GAF variables of full sample (*N* = 600) resulted in six factors is presented in Table 2. Six factors had eigenvalues>1. The first factor, with an eigenvalue of 5.327 and high loading coefficients (>0.4) for N1-5 was labeled ‘Factor-1: Negative symptoms’. The second factor, with an eigenvalue of 2.249 and high loading coefficients for N6, Current and Drop GAF scores was labeled ‘Factor-2: Global functions’. The third factor, with an eigenvalue of 1.727 and high loading factors for P5, N5, D1, D4, and G3, was labeled ‘Factor-3: Disorganized communication and behavior’. The fourth factor, with an eigenvalue of 1.268 and high loading factors for G1, G2, and G4, was labeled ‘Factor-4: General symptoms’. The fifth factor, with an eigenvalue of 1.158 and high loading factors for P1 and D2, was labeled ‘Factor-5: Odd thoughts’. The sixth factor, with an eigenvalue of 1.092 and high loading factors for P2 and P4, was labeled ‘Factor-6: Distorted cognition and perception’. Two items (P3-Grandiosity and D3-Trouble with Focus and Attention) did not load on to any factor with a loading higher than 0.4.

**Table 2:** Standardized factor loadings obtained from exploratory factor analysis, using varimax rotation, of 14 clinical items and two GAF (Global Assessment of Functioning) scores from the SIPS (*N* = 600).

Variables	Factor-1	Factor-2	Factor-3	Factor-4	Factor-5	Factor-5
P1	0.011	0.111	0.084	−0.03	0.851	0.03
P2	0.023	0.167	−0.141	0.016	0.025	0.594
P3	−0.247	0.252	0.352	−0.153	−0.186	0.24
P4	0.02	−0.021	0.148	0.059	0.117	0.688
P5	0.078	0.204	0.579	−0.128	0.141	−0.387
N1	0.762	0.262	0.121	0.051	−0.059	0.038
N2	0.701	0.357	0.053	0.237	−0.027	0.027
N3	0.834	0.089	0.284	−0.034	0.093	0.046
N4	0.828	0.1	0.207	−0.052	0.063	−0.043
N5	0.432	0.137	0.568	−0.05	0.181	−0.291
N6	0.294	0.734	0.065	0.069	0.157	0.062
D1	0.291	0.02	0.622	−0.12	0.225	0.151
D2	0.047	0.108	0.108	0.066	0.886	0.101
D3	0.051	0.34	0.318	0.245	0.031	0.259
D4	0.355	0.235	0.463	−0.113	0.046	0.029
G1	−0.049	0.049	0.073	0.798	−0.023	0.047
G2	0.073	0.074	−0.134	0.799	−0.054	−0.065
G3	0.177	−0.008	0.712	0.137	−0.029	0.03
G4	0.065	0.321	−0.061	0.618	0.228	0.252
Current-GAF	−0.295	−0.829	−0.12	−0.132	−0.097	−0.069
Drop-GAF	0.133	0.836	0.119	0.123	0.052	0.019

Notes: Factor-1: Negative symptoms (N1 social anhedonia; N2 avolition; N3 expression of emotion; N4 experience of emotions and self; N5 ideational richness). Factor-2: Global functions (N6 occupational functioning; Current-GAF; GAF drop, GAF score baseline from highest in the past year). Factor-3: Disorganized communication and behavior (P5 disorganized communication; N5 ideational richness; D1 odd behavior or appearance; D4 impaired personal hygiene; G3 motor disturbances). Factor-4: General symptoms (G1 sleep disturbance; G2 dysphoric mood; G4 impaired tolerance to normal stress). Factor-5: Odd thoughts (P1 unusual thought content; D2 bizarre thinking). Factor-6: Distorted cognition and perception (P2 suspiciousness; P4 perceptual abnormalities).

### Effect size comparison on 6 factors

**Fig. 1:**
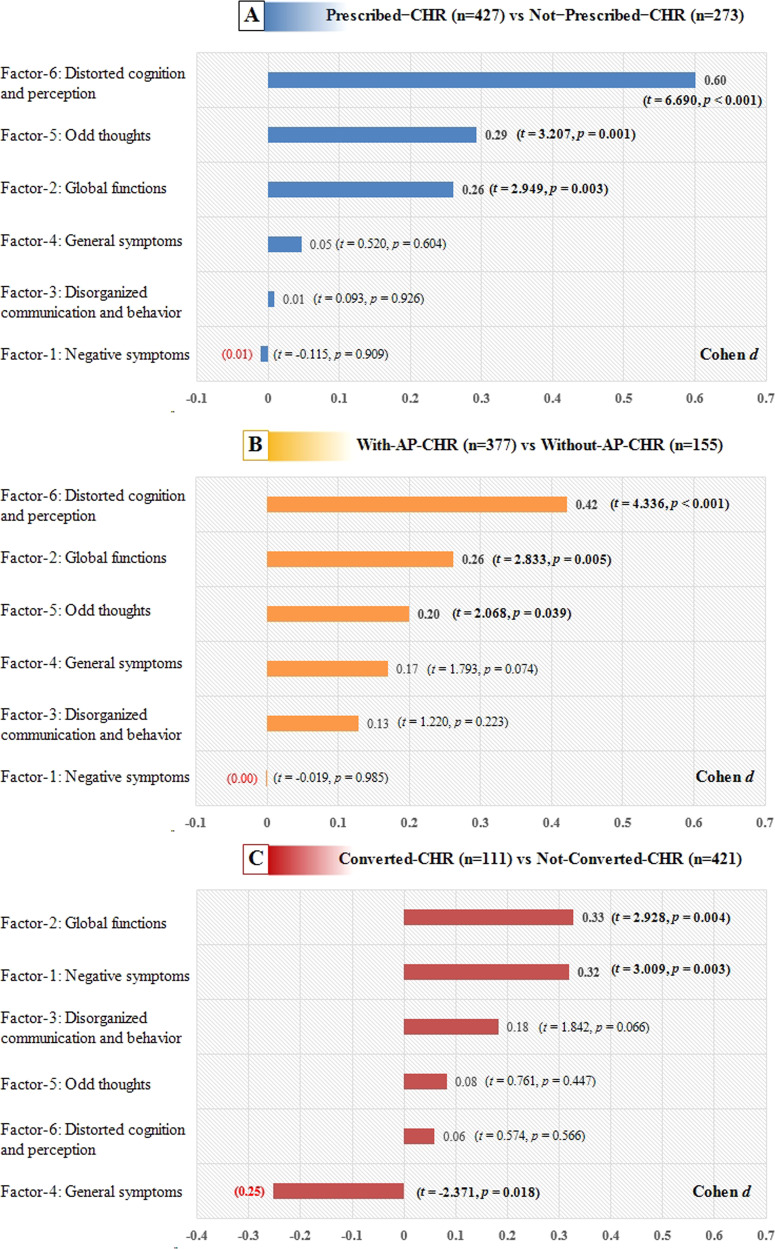
Effect sizes (Cohen *d*) for 6 factorial scores compared between prescribed-CHR and Not-Prescribed-CHR (**A**), With-AP-CHR and Without-AP-CHR (**B**), Converted-CHR and Not-Converted-CHR (**C**).

The effect sizes across the 6 factorial scores from comparisons of Prescribed-CHR and Not-Prescribed-CHR, With-AP-CHR and Without-AP-CHR, Converted-CHR and Not-Converted-CHR are presented in Fig. 1. The effect sizes on the factor-5 and 6 (related to positive psychotic symptoms) were greater and significant for comparison of prescribed-CHR and Not-Prescribed-CHR, With-AP-CHR and Without-AP-CHR. However, the effect sizes on the factor-1 (related to negative symptoms) were greater and significant for comparison of Converted-CHR and Not-Converted-CHR. Factor 2 (related to global functions) was significant in all comparisons.

### Inferencing on AP prescription from the six-factorial model

We used logistic regression to evaluate the effect of demographic (i.e. age, gender, education), and six factorial psychopathological variables on clinicians’ decisions of AP-prescription. Table 3 showed that the factor 2,5,6 (i.e. Global functions, Odd thoughts and Distorted cognition and perception) were found to be significant predictors in AP-prescription. The same factors also predicted CHR individuals’ decisions of AP-taking (Table 4). Conversion, was predicted by factor 1,2,4 (i.e. Negative symptoms, Global functions and General symptoms) (Table 5). Notably, the general symptoms (factor-4) was appeared as a protective factor in the model, meaning that the higher is the level of general symptoms the lower is the risk for conversion.

**Table 3:** Logistic regression for predicting the clinicians’ decision of antipsychotics prescription on demo8graphic and factorial variables (*n* = 600).

Predictor factor	Beta	S.E.	β	95%CI for β		Wald statistic	*P* value
Age	0.011	0.021	1.011	0.970	1.054	0.262	0.609
Gender	−0.149	0.198	0.861	0.585	1.268	0.572	0.449
Education	−0.012	0.042	0.988	0.910	1.072	0.089	0.765
Factor-1: Negative symptoms	−0.011	0.100	0.989	0.814	1.203	0.012	0.914
Factor-2: Global functions	0.305	0.097	1.356	1.122	1.640	9.921	**0.002**
Factor-3: Disorganized communication and behavior	0.046	0.099	1.047	0.863	1.271	0.216	0.642
Factor-4: General symptoms	0.023	0.095	1.024	0.849	1.233	0.060	0.806
Factor-5: Odd thoughts	0.328	0.097	1.388	1.147	1.680	11.350	**0.001**
Factor-6: Distorted cognition and perception	0.647	0.107	1.910	1.550	2.354	36.869	**<0.001**

Notes: Beta is the regression coefficient. S.E. is the standard error. 95% CI is the estimated 95% confidence interval for the corresponding parameter. β is the standardized regression coefficient.

*p* values that are statistically significant are shown in bold.

**Table 4:** Logistic regression for predicting the CHR individuals’ decision of antipsychotics assumption on demographic and factorial variables (*n* = 532).

Predictor factor	Beta	S.E.	β	95%CI for β		Wald statistic	*P* value
Age	−0.014	0.023	0.986	0.942	1.031	0.391	0.532
Gender	0.029	0.205	1.030	0.689	1.538	0.021	0.886
Education	0.007	0.045	1.007	0.923	1.099	0.022	0.881
Factor-1: Negative symptoms	−0.011	0.102	0.989	0.810	1.208	0.011	0.916
Factor-2: Global functions	0.308	0.105	1.360	1.107	1.672	8.564	**0.003**
Factor-3: Disorganized communication and behavior	0.147	0.108	1.158	0.938	1.431	1.863	0.172
Factor-4: General symptoms	0.167	0.100	1.182	0.971	1.438	2.783	0.095
Factor-5: Odd thoughts	0.227	0.101	1.255	1.030	1.529	5.058	**0.025**
Factor-6: Distorted cognition and perception	0.433	0.109	1.542	1.246	1.908	15.841	**<0.001**

Notes: Beta is the regression coefficient. S.E. is the standard error. 95% CI is the estimated 95% confidence interval for the corresponding parameter. β is the standardized regression coefficient.

*p* values that are statistically significant are shown in bold.

**Table 5:** Logistic regression for predicting the conversion to psychosis on demographic and factorial variables (*n* = 532).

Predictor factor	Beta	S.E.	β	95%CI for β		Wald statistic	*P* value
Age	−0.041	0.027	0.960	0.911	1.011	2.390	0.122
Gender	−0.291	0.229	0.747	0.477	1.171	1.617	0.203
Education	0.082	0.052	1.085	0.980	1.201	2.472	0.116
Factor-1: Negative symptoms	−0.326	0.110	0.722	0.582	0.895	8.825	**0.003**
Factor-2: Global functions	−0.333	0.117	0.717	0.570	0.902	8.075	**0.004**
Factor-3: Disorganized communication and behavior	−0.176	0.099	0.839	0.690	1.019	3.140	0.076
Factor-4: General symptoms	0.260	0.116	1.297	1.034	1.627	5.062	**0.024**
Factor-5: Odd thoughts	−0.140	0.113	0.870	0.697	1.085	1.530	0.216
Factor-6: Distorted cognition and perception	−0.119	0.114	0.888	0.710	1.111	1.081	0.299

Notes: Beta is the regression coefficient. S.E. is the standard error. 95% CI is the estimated 95% confidence interval for the corresponding parameter. β is the standardized regression coefficient.

*p* values that are statistically significant are shown in bold.

## Discussion

### Summary of findings

This paper aims at investigating the complex, yet clinically central, interconnection of clinicians’ intention of antipsychotic prescription, CHR individuals’ intention of assuming antipsychotic therapy and the psychopathological risk factors for conversion to psychosis in a large cohort. Four major findings were obtained: First, severe baseline positive symptoms such as thought and perception abnormality and impairments of global functions predicted the AP prescriptions by clinicians for CHR individuals. Second, CHR individuals’ decision of AP-taking is associated to the same factors motivating clinicians’ AP prescription (i.e. positive symptoms and global functions). Third, CHR individuals exhibiting more severe negative symptoms (such as social anhedonia and impairments of global functions) at baseline were more likely to convert to psychosis in the following 2 years. Finally, while both clinicians and CHR individuals appear focused on global functions and the management of positive symptoms by AP treatment already in the very early phase of psychosis, an important empirical predictor of conversion, i.e. negative symptoms, is less likely to be improved by AP treatment and requires specific treatment attention.

In sum, while clinicians’ prescription and patients’ assumption largely cohere with respect in the target psychopathological features, namely positive symptoms and global functions, these overlap only partially with respect to the prevention aim. Indeed, besides global functions also negative symptoms were found to play an important role in determining the actual risk of transition to psychosis in our CHR sample and they are allegedly only marginally affected by AP treatment. Therefore, the specific area of prodromal negative symptoms would require sustained therapeutic attention if we want to improve outcomes.

### Clinicians’ considerations of AP prescriptions

We found that baseline positive symptoms were the significant predictors of AP prescriptions by clinicians. Specifically, CHR individuals with the higher severity level of symptoms of distorted cognition and perception (suspiciousness and perceptual abnormalities) and odd thoughts (unusual thought content and bizarre thinking) were more likely to be treated with AP. It’s not surprising since the essence of the CHR identification was based on attenuated positive symptoms, such as delusions and hallucinations^9,10^. The effectiveness of AP on positive symptoms in patients with psychosis^11,12^ and CHR individuals^13–15^ had been widely reported. Our results suggest that clinicians follow a rational, symptom oriented clinical strategy, which becomes fully visible adopting a dimensional perspective (such as the one derived from EFA factors) beyond the canonical categorical stratification of CHR subgroups. Our results suggest that clinicians should adopt a longer-term vision, beyond the purely on-demand symptomatic treatment. There is increasing evidence that non drug treatments such as cognitive-behavioral therapy^16^ for psychosis may be effective for individuals with attenuated positive symptoms. There is another realistic reason why AP is widely prescribed by our clinicians is the lack of non-drug treatment for CHR populations. Therefore, the development of diversified early interventions may help to improve the widespread use of AP.

### CHR individuals’ considerations of AP taken

The decision from CHR individuals (or their family members) to take AP seems consistent with the clinicians’ prescriptions. The assumption of AP was most significantly predicted by the suspiciousness and perceptual abnormalities symptoms (factor-6, see Fig. 1). Previous studies^17,18^ found that perceptual abnormalities can lead to the increase of anxiety and depression symptoms of CHR individuals, which is more likely to attract the attention of patients and their families. On the other hand, perceptual abnormalities symptoms are easier for clinicians and patients to identify as relatively abnormal. Also, when perceptual abnormalities occur, people often think that the psychosis has begun, which makes the use of antipsychotics a reasonable option. However, perceptual abnormalities such as hallucinations, especially isolated perceptual symptoms may play a role of “nonspecific/clinical noise” for predicting psychosis in CHR phase^19^ or normal variations among the general population^20,21^. Whether it is suitable for use as an indication of AP has not been determined.

Slightly different from clinicians’ second reason for clinicians’ decisions of AP-prescription (odd thoughts), CHR individuals’ decisions of AP-taking is also predicted by global functions. This might be related to the fact that usually thought symptoms are relatively ego-syntonic and less worrisome for patients that for clinicians. For CHR individuals as well as for their caregiver, on the contrary, changes in functioning, such as academic and inter-peer performance, is of obvious impact and immediately perceivable^22^. Deflections in Global functions is typically the driving force for the CHR individuals and their caregivers to seek for professional help to recover^23^, and AP taken might be mostly motivated by the hope of gaining previous functional levels back again. On the other side, negative symptoms such as observed withdrawal or pro-active isolation are more easily attributed to relatively typical adolescent behavior^24^, and might be perceived as less relevant for treatment seeking as well as for need-based treatment.

### Risk factors for conversion to psychosis

A growing body of research has demonstrated that negative symptoms are significant predictor of conversion to psychosis in CHR population^25,26^. In current study, CHR individuals with severe negative symptoms at baseline were more likely to convert to psychosis. In contrast, the positive symptoms that had been targeted and treated by AP were not even significant in our prediction model. While much effort has been dedicated to positive symptoms in the prospective of CHR identification and conversion^27^, negative symptoms (and partly global functions), on the other side, may be largely neglected in clinical practice and research. Bearing in mind the little effectiveness of AP on negative symptoms^28^ and the risk that some of their side effects may aggravate the negative symptoms^29,30^, the early use of AP for the purpose of effective prevention of psychosis should be particularly cautious^6,31^. Future study of newer approaches targeting negative symptoms in CHR^32^, such as *N*-methyl-D-aspartate receptor modulator interventions (glycine and d-serine), cognitive remediation therapy, and family therapy should be undertaken.

Interestingly, in this study, the functional impairment is not only the focus of the three parties, but also the contradiction. This may be related to the special status that function is related to both positive and negative symptoms. From the perspective of CHR individuals, the most important concern is to restore the function. From the perspective of clinicians, they hope to improve the function by controlling the positive symptoms with AP prescriptions. However, in the process of early psychosis, on the one hand, the function improves with the improvement of positive symptoms by AP treatments, on the other hand, it deteriorates with the deterioration of negative symptoms and the influence of AP treatment itself. Consistent with previous findings, poor function^33^ and adjustment^34^ in CHR individuals was associated with an increased risk of conversion to psychosis. On the other hand, there is research evidence^25,26,35^ that CHR individuals with lower levels of negative symptoms and higher levels of social functioning are more likely to recover symptomatically.

Notably, the general symptom factor revealed a protective effect with respect to the conversion to psychosis. Specifically, CHR individuals with higher severity of general symptoms had a significantly lower conversion risk compared with CHR individuals with lower levels of general symptoms. However, the mechanism behind such apparent protective role is not entirely clear. Indeed, first there are inconsistencies in the literature^36,37^, which found that baseline mood disturbance is associated with poor prognosis but had no effect on risk of transition to full psychosis. Also, previous studies had not included other variables, such as dysphoric mood, as a potentially relevant factor for predicting psychosis, although dysphoric mood has been found to be related to clinical caseness in CHR cohorts rather than to progression towards psychosis^38^. On a clinical level, however, general symptoms are better understood as a cloud of relatively unspecific symptoms that certainly motivate referral but are not specifically predictive of the progression towards psychosis.

### Strengths and limitations

The main strengths of this study are its longitudinal, prospective design with determined clinical outcome, stratified clinicians’ decision (AP-prescription) and CHR individuals’ decision (AP-taking) in the analysis, large sample size of drug naïve CHR individuals and examination of the SIPS factor structure. Furthermore, all included participants were AP-naïve prior to the moment of CHR assessment as per inclusion criteria, which substantially mitigates some potential, AP-related prognostic confounders (e.g. the risk of including pharmacologically-attenuated first episode psychosis within the CHR sample)^39^, Some limitations of our study, however, merit comment. First, this CHR cohort was surveyed naturalistically, and various medications other than AP (such as antidepressants) that CHR individuals might have assumed with varying compliance during the follow-up period may have an impact the clinical outcomes. However, a major focus of this study is the decisions of CHR individuals on AP-taking rather than the process of AP usage. The information of decision making on AP-taking from CHR individuals is accurate which had been carefully recorded according to CHR individuals and their caregivers during multiple follow-up. A second limitation is the lack of more detailed information on the background of our prescribing clinicians, including career seniority, educational background, field of expertise, familiarity with CHR assessment and treatment standards, etc. Those variables may impact their decision of AP prescriptions. A third limitation is the lack of data on the compliance of CHR individuals, the inferences of their caregivers, even their financial situation and medical insurance situation may inference the CHR individuals’ decision on AP taken. Although we performed tripartite checks-involving the CHR individuals, their family members, and clinician reports plus medical records to confirm and quantify the AP treatment details, our approach was less accurate than other strict methods, such as pill counts and self-report.

## Conclusions

There are intuitive gaps between the focus of clinicians’ AP prescriptions, expectations of CHR individuals’ taking AP, and the clinical symptom-related risk factors predicting conversion to psychosis. With the exception of global functions are recognized as central by clinicians and CHR individuals and play a clear role in increasing the transition risk, clinicians and CHR individuals’ perspectives converge on positive symptoms (which in our sample did not seem to pay a central role in predicting later conversion) and pay definitely less attention to negative symptoms (which proved a robust predictor of conversion to psychosis). It is clear that a realignment in the understanding of AP usage is necessary, and that important risk predictors of conversion, such as negative symptoms, largely fall outside the therapeutic spectrum of AP. However, treating negative symptoms in CHR remains an important yet elusive target: indeed, a recent network meta-analysis^32^ which reviewed 11 treatment approaches for negative symptoms in CHR, found that effectiveness did not reach statistical significance for any of the treatments at stake. Thus developing novel interventions to decrease the burden of negative symptoms in CHR youth is a clear priority for preventive purposes^16,40,41^. On pragmatic side, although our result confirms that AP prescription in CHR is rationally associated with specific psychopathological profiles, further education and training on rational use of AP may help improving prescriptive appropriateness and ultimately improve care and management of CHR individuals. Furthermore the results indirectly suggest that going beyond CHR categorical strata towards a more detailed dimensional approach^42^ (such as the current factorial decomposition of SIPS/SOPS) is feasible^43^ and could be an important move for a more personalized and precise treatment decision making.

## Methods

### Sample, design and setting

The data used for this study was derived from the ShangHai At Risk for Psychosis-extended(SHARP-extended) program. The methodology and study design of the SHARP-extended have been described in detail elsewhere^44–47^. Six hundred CHR individuals were recruited between 2016-2019 year at the Shanghai Mental Health Center(SMHC) in China(Clinical trials.gov identifier NCT04010864)^48^. The Research Ethics Committees at the SMHC approved this study. All participants gave written informed consent at the recruitment stage of the study. Subjects younger than 18 years of age had their consent forms signed by their parents, but they also gave written informed consent themselves. Patients had to fulfill at least one of the prodromal syndrome criteria: (1) brief intermittent psychotic syndrome (BIPS), (2) attenuated positive symptom syndrome (APSS), or (3) genetic risk and deterioration syndrome (GRDS). Inclusion criteria were: (i) under age of 45 years old; (ii) individuals younger than 18 years old had to be accompanied by either a parent or legal guardian; (iii) capacity to provide informed consent or assent if under 18; (iv) must have completed at least 6 years of primary education; and (v) be psychotropically naïve at the moment of CHR assessment. Exclusion criteria were: (i) severe somatic diseases, for example, pneumonia, cancer or heart failure, (ii)intellectual disability, or (iii) had a history of drug (such as methamphetamine) abuse or dependence.

The referral medical system does not exist in China, so patients are generally free to choose hospitals and doctors. In the first visiting, CHR individuals were referred to the participating the naturalistic follow-up study, which research procedure was independent of the routine clinical treatment. The study does not interfere with clinicians’ prescription decisions and CHR individuals’ decisions of AP-taking. Of the total of 600 CHR individuals completed the baseline assessment, 68 individuals have not reached the end of follow-up, remained 532 individuals who completed 2-year follow-up, reassessed by telephone or by face-to-face interview every 6 months using the Structured Interview for Prodromal Syndromes (SIPS)^49,50^.

The SMHC is the largest outpatient mental health clinic that offers medication management and psychotherapy in China. The outpatients come from different parts of the country. The SMHC is a comprehensive psychiatric hospital in Shanghai that sees over 1,000,000 outpatients per year. The participants were recruited from the department of the Shanghai Psychotherapy and Psychological Counseling Center (SPCC) at the SMHC. There are approximately 1000 professional staff who provide care for the patients at the SMHC. Among them, 258 are psychiatrists and psychologists and 541 are psychiatric nurses, along with other support staff. The SPCC provides comprehensive clinical services, including psychological assessment and counseling as well as medical management. Patients come seeking help for issues ranging from general psychological problems (e.g., interpersonal adaptation, marriage and learning difficulties) to more severe psychological disorders and mental illnesses (e.g., schizophrenia and bipolar disorder).

### Assessments

CHR status and transition to psychosis were evaluated using the SIPS. The SIPS consists of 19 items that assess four symptom domains: positive symptoms (scales P1–P5: P1 unusual thought content; P2 suspiciousness; P3 grandiosity; P4 perceptual abnormalities; and P5 disorganized communication), negative symptoms(scales N1–N6: N1 social anhedonia; N2 avolition; N3 expression of emotion; N4 experience of emotions and self; N5 ideational richness; and N6 occupational functioning), disorganized symptoms (scales D1–D4: D1 odd behavior or appearance; D2 bizarre thinking; D3 trouble with focus and attention; and D4 impaired personal hygiene), and general symptoms (scales G1–G4: G1 sleep disturbance; G2 dysphoric mood; G3 motor disturbances; and G4 impaired tolerance to normal stress). Functioning was assessed with the global assessment of function (GAF) measuring the participants’ global psychological, social, and occupational functioning. The drop in GAF scores was used for assessing the functional deterioration (i.e. the GAF score relative to 12 months prior) in the SIPS interview. Conversion to psychosis was defined using the POPS (presence of psychotic symptoms in SIPS) criteria. The conversion was defined as the development of at least one psychotic level symptom (rated ‘6’on the SIPS positive symptoms scale) with either sufficient frequency or duration, or occurring at least an hour a day on average over four days a week for at least longer than 16 hours.

### Medication prescription and exposure

The AP prescription was given by clinicians based on the routine outpatient service. All the participants had no previous psychiatric drug history for mental disorders. The information of clinicians’ decisions on AP-prescription was recorded in the SMHC’s electronic information system. Current study is naturalistic follow-up investigation without any extra intervention or financial remuneration. The CHR individuals’ decisions on AP-taking was recorded by researchers during the follow-up assessments and confirmed by their family members and verified using clinician reports and medical records. Medication usage information systematically record including the types of antipsychotics, drug response, reduction/withdrawal time. In current study, for those CHR individuals who had taken AP for at least 2 weeks^6^ were classified as a group of CHR individuals who had made a decision on AP-taking. Notably, the proportion of CHR individuals who decided to take AP in agreement with their clinicians’ in the first visit is relatively low (i.e. accounting for about 40%). Even among CHR individuals who were confirmed to the prescription of AP in the follow-up, the types and methods of AP-assumption were mostly (about 90%) different from the initial prescription at the first visit. Therefore, CHR individuals’ decision on AP-taking is not a mere consequence of the decision of prescription from clinicians.

### Statistical analysis

CHR individuals were grouped according to baseline AP-prescription (reflecting clinicians’ decision, Prescribed-CHR vs. Not-Prescribed-CHR), AP-taking during the follow-up (reflecting CHR individuals’ decision, With-AP-CHR vs. Without-AP-CHR) and conversion to psychosis (reflecting the natural process of psychosis, Converted-CHR vs. Not-Converted-CHR). Quantitative variables are expressed as mean ±S.D., while qualitative variables are presented as frequencies (%). The two groups were compared using *χ*^2^ tests for comparisons of categorical variables and independent *t* tests for comparisons of continuous variables. Comparisons between Prescribed-CHR and Not-Prescribed-CHR groups, With-AP-CHR and Without-AP-CHR groups, Converted-CHR and Not-Converted-CHR groups were conducted separately. Effect sizes were calculated using Cohen’s *d* for mean comparisons^51^. The exploratory factor analysis was applied using the principal components analysis and varimax rotation. The number of factors retained in the analysis was based on retaining factors that accounted for >10% of the common variance as well as interpretability. Then, using the factor loading coefficients, we calculated the estimated factor scores for each factor for all CHR individuals. A multiple logistic regression analysis was conducted to predict clinicians’ decisions of AP-prescription, CHR individuals’ decisions of AP-taking, and conversion to psychosis using age, gender, education, and estimated factor scores as predictors.

## Data Availability

All relevant data are available from the authors upon reasonable request.
